# Knowledge and Training Needs in Nosocomial Infection among Hospital Staff in the City of Kielce, Poland: A Cross-Sectional Study

**DOI:** 10.1155/2024/9243232

**Published:** 2024-04-09

**Authors:** Kamila Fortunka, Agnieszka Strzelecka, Grzegorz Król, Paulina Paprocka, Angelika Mańkowska, Agata Lesiak, Urszula Karpeta, Slawomir Okła, Jakub Spałek, Szczepan Kaliniak, Ewelina Piktel, Maciej Karasiński, Bonita Durnaś, Robert Bucki

**Affiliations:** ^1^Department of Microbiology and Immunology, Institute of Medical Science, Collegium Medicum, Jan Kochanowski University of Kielce, IX Wieków Kielc 19A, 25-317, Kielce, Poland; ^2^Institute of Health Science, Collegium Medicum, Jan Kochanowski University of Kielce, IX Wieków Kielc 19A, 25-317, Kielce, Poland; ^3^Institute of Medical Science, Collegium Medicum, Jan Kochanowski University of Kielce, IX Wieków Kielc 19A, 25-317, Kielce, Poland; ^4^Department of Otolaryngology, Head and Neck Surgery, Holy-Cross Cancer Center, Artwińskiego 3, 25-734, Kielce, Poland; ^5^Department of Medical Microbiology and Nanobiomedical Engineering, Medical University of Białystok, Jana Kilińśkiego 1, 15-089, Białystok, Poland

## Abstract

**Introduction:**

Nosocomial infections are an integral part of health care services, posing a threat to both patients and medical staff. The duty and role of nursing staff is to prevent nosocomial infections in every hospitalized person. *Material and Methodology*. The study involved 635 nurses working in various surgical and conservative wards. The technique used was the author's questionnaire, which contained 30 questions and was divided into three components.

**Results:**

The level of knowledge among the surveyed nurses was at a sufficient level for more than half of the total tested population, and its level was influenced by two variables: the specialization held and the level of education. Nurses working in medical wards have a higher level of knowledge in the area of basic concepts related to nosocomial infections, and people who use specialist medical literature and participate in specialist courses have a sufficient level of knowledge. The shortest time since the last training results in a higher level of knowledge and a higher level of knowledge in the area covering the basic concepts of nosocomial infections. The most frequently selected issues on which nurses would like to expand their knowledge were post-exposure procedures and methods of monitoring nosocomial infections.

**Conclusions:**

The knowledge of the nursing staff in the field of nosocomial infections is diverse, and its main determinants are specialization, education, and age. A sufficient level of knowledge among the respondents is conditioned primarily by the use of specialist literature and participation in specialist courses, which determine both the scope and area of knowledge on nosocomial infections.

## 1. Introduction

Nosocomial infections are a challenging problem in modern medicine, occurring as adverse events in connection with the provision of health services [1]. They are a therapeutic encounter, an epidemiological problem, but also an economic problem due to the high costs mostly associated with prolonged stays of patients in hospitals [2]. The hospital environment is a favorable place for the occurrence and spread of infections both in the group of patients and medical staff [3]. The literature on the subject emphasizes the long-known fact that nosocomial infections are an inseparable part of the treatment process, while the hands of the staff are the most important vector of transmission of microorganisms in the group of hospitalized patients. Already in the 1990s, the Centers for Disease Control and Prevention (CDC) showed that proper hand hygiene is the most effective, simplest, and, at the same time, cheapest way to prevent nosocomial infections [4]. Nosocomial infections may occur among patients treated both in conservative and surgical wards [5]. Frequently, nosocomial infections undermine the results of treatment for the underlying disease and prolong the period of hospitalization for the patient, thus creating a risk of losing their job and adversely affecting their mental health. Nosocomial infections can also lead to the death of the patient, which may result in serious legal consequences [6]. The effects of nosocomial infections affect not only the individual patient but also the entire society by affecting the cost of health premiums [7].

Reducing the frequency of nosocomial infections is a complex process, depending on several key elements: knowledge of nosocomial infections, knowledge of procedures and their observance, proper hospital hygiene, which translates into proper interruption of pathogen transmission, as well as properly applied rules of asepsis, which is crucial in preventing infections in people who underwent surgical procedures [8]. These activities are labour-intensive, tedious, and oblige us to constantly recreate the correct patterns of preventive behavior [9]. Medical personnel should have optimal qualifications, both theoretical and practical, regarding the procedures performed [10]. The largest professional group in hospitals, and at the same time the one that has the most frequent contact with patients, is the nursing staff. Therefore, they are required to take a responsible attitude at every stage of patient care, i.e., during the diagnosis of the disease, treatment planning, as well as the entire diagnostic and therapeutic process. Hygiene, asepsis, antisepsis, and work organization procedures functioning in hospitals must be very well known and performed with due diligence. Nurses' knowledge of nosocomial infections and adherence to procedures while performing nursing activities is an overriding factor that must translate into conduct in their daily work [11, 12].

Many studies indicate that there are significant gaps between scientific knowledge in the field of nosocomial infections and its practical implementation in everyday hospital practice. Another problem is the failure to comply with procedures that prevent nosocomial infections, which leads to the health risks of both hospitalized patients and medical personnel [13]. Nursing staff should have extensive knowledge of nosocomial infections, especially in the field of prophylaxis, and knowledge and competences should be constantly consolidated, updated, and based on scientific evidence [14]. Education focusing only on the transfer of theoretical knowledge is not very effective and insufficient to change behavior. Prevention and control of nosocomial infections is most effective with a multimodal strategy [15]. Indeed, a multimodal strategy to improve hand hygiene compliance was initially approved and released by WHO in 2009. The approach uses educational tools and programs, supervision and control, active staff participation, and leadership commitment to infection control [16].

In order to acquire the appropriate skills for the prevention and control of nosocomial infections, nursing staff must undergo appropriate education and training that combine theory with clinical practice. Healthcare facilities must ensure that education correlates with the current needs of the nursing staff. Nursing staff should have time set aside for training and educational events and should be supported by their employers in applying their knowledge in practice. Internal training should be carried out by qualified persons with medical education, experts in the field of nosocomial infections, and nurses who understand the problems and needs of nurses in this area [14].

### 1.1. The Aim of Study

The main objective of the study was to assess the state of knowledge and education needs of nursing staff on nosocomial infections among nurses working in hospitals in the city of Kielce.

## 2. Materials and Methods

### 2.1. Variables and Their Pointers

The independent variables and their indicators were socio-demographic factors, i.e., gender (female, male), age (under 30, 31 to 39, 40 to 49, over 50), education (medical secondary school, medical vocational, higher professional bachelor of nursing, higher master's degree in nursing), work experience (up to 10 years, from 10 to 20 years, over 20 years), specialization (yes or no), and place of work (non- or surgical ward). The dependent variable in the conducted study was the knowledge of the nursing staff about nosocomial infections, while the indicator for the adopted variable was the respondents' answers to the questions included in the questionnaire. The ranges for the variables were selected based on the analysis of similar studies on this topic.

### 2.2. Population and Sample of the Study

The number of nursing staff in the Świętokrzyskie Voivodeship is 5,290 people. There are 20 hospitals in the entire province. These are 1st, 2nd, and 3rd degree hospitals, oncology and pulmonology hospitals, and nationwide hospitals. There are five hospitals in the city of Kielce. Two first-level hospitals: Kielce Hospital of St. Aleksandra -Limited Liability Company and Świętokrzyskie Center for Mother and Newborn -Specialist Hospital in Kielce. One tertiary hospital is the Provincial Combined Hospital in Kielce. There is also one state hospital: the Independent Public Health Care Center of the Ministry of Interior and Administration in Kielce, and one oncology hospital: the Świętokrzyskie Oncology Center. The list of all hospitals and the number of nurses in the city of Kielce was prepared on the basis of data from the National Health Fund. The list of all hospitals in the Świętokrzyskie Voivodeship was prepared on the basis of data from the National Health Fund.

The research was carried out in all hospitals in the city of Kielce, except the Świętokrzyskie Center for Mother and Newborn - Specialist Hospital in Kielce, because mainly midwives work in this hospital. Among all medical staff working in hospitals, the focus was exclusively on nurses because nursing staff have the most frequent contact with patients and play a key role in the prevention of nosocomial infections.

Participation in the study was offered to all nurses who worked in the hospitals participating in the study. When selecting the sample for the study, the sample size calculator of the statistical program STATISTICA version for Windows 13.1 TIBCO Software Inc. was used. – StatSoft, Poland, with a 95% confidence interval. This made the sample representative. Based on data on the number of people working in the city of Kielce, the minimum group of nursing staff that should be included in the study was 311 people.

### 2.3. Research Methods and Tools

In order to achieve the goal set in the planned study, the diagnostic survey method was used. The survey questionnaire was developed based on the analysis of many similar studies, in which the researchers also used their own questionnaires to assess the knowledge of medical staff. Most research works on this problem are based on proprietary surveys, and such a survey was also used in the presented study [17–19].

The technique used was the author's questionnaire, which contained 30 questions and was divided into three components. The first part contained 6 questions and concerned sociodemographic data; the second part included 20 questions related to issues testing knowledge about nosocomial infections, while the last part included 4 questions concerning training in acquiring knowledge about nosocomial infections.

The tool prepared in this way was validated. Validations were carried out before the actual examination on a group of 294 nurses. Cronbach's alpha was 0.751, which proves the reliability of the tool - a self-designed questionnaire, which included questions on the state of knowledge in the field of nosocomial infections.

### 2.4. Data Collection Method

Based on data on the number of nurses working in the city of Kielce, the minimum number of respondents that should be included in the study was determined to be 311 people, but ultimately a larger research group of 635 people was studied. This resulted in the surveyed sample being representative. The surveys were distributed and collected from March to September 2022, and the questionnaire was distributed to various departments by the authors.

#### 2.4.1. Data Analysis

While examining the state of knowledge and the need for education of the nursing staff on nosocomial infections, 20 statements were identified that determined its overall indicator. The answers given by the respondent were required to be classified as correct or incorrect. Each correct answer was assigned a value of 1, and 0 points for an incorrect answer. Then, the points were summed up, and the maximum number of possible scores was 20.

The statistical methods used in the work depended on the types of variables analyzed. For qualitative variables, i.e., gender, type of department, specialization, age, education, or work experience, the distribution (*n*) and frequency (%) are given, and to verify the independence of variables determining the level and state of knowledge, the *χ*^2^ test was used, or, in the case of a small number, the least numerous classes (*n* < 5) *χ*^2^ test based on maximum likelihood functions (NW). The *χ*^2^ independence test is based on the comparison of observed and expected numbers (the expected numbers are determined assuming that the null hypothesis is true). For quantitative variables (raw values of the level of knowledge), location measures are provided: median (Me), lower quartile (Q1), and upper quartile (Q3), and the lowest (min) and highest (max) values of the examined parameter are indicated.

Searching for an answer to the research problem, how the level of knowledge regarding hospital infections develops (numerical values - raw indicator) depending on selected sociodemographic parameters or the type of ward (medical or surgical), differences in the distribution of the examined parameter were verified using the nonparametric *test Mann–Whitney U test*(when comparing two independent groups) or using *the Kruskal–Wallis ANOVA* rank test (when comparing more than two independent groups). Subsequently, if the null hypothesis was rejected in favor of an alternative hypothesis for these tests (statistic value of a given test *p* < *α* for more than two independent groups), multiple comparisons of the mean ranks for all samples (post hoc) were performed to determine a pair of variables for which the distribution of the examined parameter was statistically significantly different. *Spearman's rho* correlation analysis was used to determine the relationship between ordinal variables (qualitative variables) and raw numerical values between the studied variables. Logistic regression analysis was used to determine the predictors influencing the level and sources of the respondents' knowledge and participation in specialized courses. The construction of the model was preceded by a preliminary selection of predictors by assessing their quality using Crammer's V coefficient. At this stage, some of the predictors were rejected, and then the sequential construction of the logistic regression model began. For this purpose, forward stepwise regression was used, and the significance of the difference between subsequent sequentially built models was assessed using the LR test (likelihood ratio). The goodness of fit of the model was verified using the Hosmer-Lemeshow test. Then, an ROC curve was constructed for the same pairs of variables, which was used to assess the compliance of the studied factors resulting from the model with the actual indications. The area under the ROC curve was calculated, denoted as AUC (area under curve), which is a measure of the goodness of the model. The Youden Index was used to determine the cut-off point for raw numerical values and to determine the sufficient level of knowledge of respondents regarding nosocomial infections.

The selected significance level of *α* = 0.05 was adopted in the work. The data was collected in an Excel spreadsheet belonging to the MS Office package by Microsoft. Statistical analysis was performed in STATISTICA version for Windows 13.1 TIBCO Software Inc. – StatSoft, Poland. Data are presented in the form of tables and figures.

#### 2.4.2. Characteristics of the Study Group

Among the respondents, the majority were women (94.00%), aged up to 30 years (32.30%), and had master's degrees (58.40%). More than half of the respondents had no specialization (60.00%), their work experience was up to 10 years (43.50%), and a larger number of nursing staff worked in surgical departments (55.00%) (Table 1).

## 3. Results

### 3.1. Sociodemographic Factors Affecting the Level of Knowledge among the Respondents

By examining the state of knowledge and the need for education of nursing staff on nosocomial infections, 20 statements were identified in three thematic areas that determined its overall index. The first area covered the basic concepts of nosocomial infections and consisted of 6 factors. The second area concerned issues in the field of infection/microbiology with particular emphasis on microbiological diagnostics, and also included 6 factors. The third and last area concerned the methods of preventing nosocomial infections and included 8 elements that influenced the assessment of the state of knowledge.

The answers given by the respondents had to be classified as correct or incorrect. Each correct answer was assigned a value of 1, and 0 for an incorrect answer. Then, the points were summed up, and the maximum number of possible points received was 20. In this approach, only correctly given answers were assessed and interpreted, which allowed for a good differentiating power of the respondents into groups with a different level of knowledge in the discussed area.

Subsequently, it was determined to what extent sex, age, education, specialization, and work experience affect the level of knowledge of nurses regarding nosocomial infections.

Comparisons of raw numerical values were made for selected sociodemographic variables. It was found that the level of knowledge about nosocomial infections was affected by the specialization, education level, and age of the surveyed nurses (Table 2).

On the basis of the raw numerical results, the general index of knowledge of the subjects regarding nosocomial infections was normalized. For this purpose, a dichotomous variable (0, 1) was created, where 0 means an insufficient level of knowledge and 1 means a sufficient level of knowledge.

**Figure 1:**
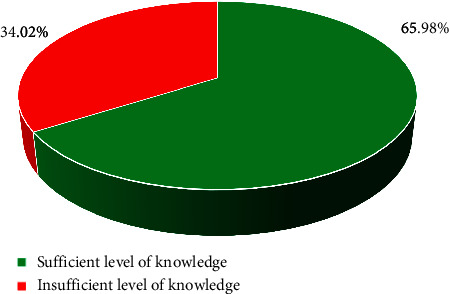
The level of knowledge of the surveyed nurses regarding nosocomial infections normalized results.

The standardized qualitative variable defined in this way allowed us to conclude that the insufficient level of knowledge was present in 216 (34.02%) and sufficient in 419 (65.98%) of the examined nurses (Figure 1).

**Figure 2:**
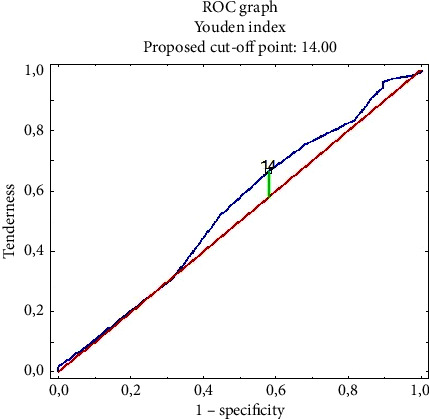
Youden index for the raw indicator of knowledge in the field of nosocomial infections in the group of surveyed nurses.

To define the state of knowledge among the surveyed nurses, the Youden Index was used (AUC = 0.537, CL 95% 0.438–0.636). The cut-off point obtained for the study group, resulting from raw scores is 14 points out of 20 possible (which is 70.00% of correct answers) (Figure 2).

The assessment of knowledge in the field of nosocomial infections (a dependent variable in the logistic regression model) was defined as a dichotomous variable with two variants: sufficient [1] and insufficient (0). The construction of the model was based on forward-step regression, and the significance of the difference between successive, sequentially built models was assessed using the LR test (likelihood ratio).

Based on the estimated logistic regression, it can be concluded that the chance of having higher knowledge in the field of nosocomial infections is 1.5 times higher among nurses with specialization (OR = 1,489; 95% CI: 1,050–2,112; *p*=0.026) than among respondents who do not have additional competences. Another determinant is the level of education - the chance of having sufficient knowledge about nosocomial infections decreases when the respondents graduate from medical high school (OR = 0.415; 95% CI: 0.294–0.998; *p*=0.004) and medical vocational studies (OR = 0.532; 95% Cl: 0.294–0.998, *p*=0.049). In the case of respondents with vocational education (Bachelor of Nursing), the level of knowledge is at a similar level compared to people with higher education (Table 3).

**Figure 3:**
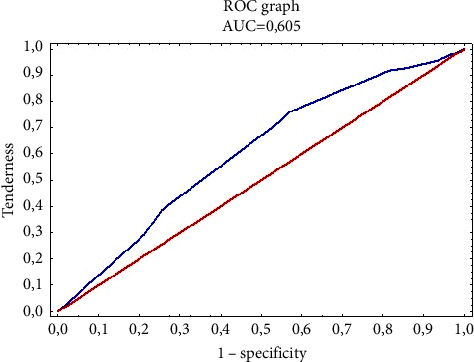
The ROC curve plot for the logistic regression model is moderately fitted to the predictors, i.e., education and specialization possessed, affecting the level of knowledge of the surveyed nurses in the field of nosocomial infections.

The value of the Hosmer–Lemeshow statistic is 2,442, with a value of *p*=0.295, which proves a significant fit of the logistic regression model. Based on the analysis of the area under the ROC curve, it can also be concluded that the model is moderately fit to the data (area is AUC = 0.605) (Figure 3) and has a moderate predictive power resulting from the obtained plots of sensitivity and specificity (specificity) for various levels probabilities.

### 3.2. Sources of Knowledge Affecting the Level of Knowledge among the Respondents

The level of knowledge among the respondents is affected by such factors as the level of education or having additional competences in the field of specialization. Factors that can also modify (differentiate) it include internal training in the workplace, scientific conferences, specialist courses, webinars and online training, specialized medical literature, and media such as television, the Internet, and nonmedical press.

Using logistic regression, it was determined which sources had the greatest impact on the level of knowledge of the respondents (dichotomous variable) regarding hospital infections. For this purpose, forward stepwise regression was used in accordance with the procedure described when creating the model in the previous section.

Based on the estimated logistic regression, it can be concluded that the chance of having sufficient knowledge in the field of nosocomial infections is 1.6 times higher among nurses who use specialist medical literature (OR = 1,612; 95% CI: 1,068–2,432; *p*=0.023) and take part in specialist courses (OR = 1,613; 95% CI: 1,007–2,581; *p*=0.047). This type of extension (supplementation) of professional competences determines a higher standardized knowledge index (Table 4).

**Figure 4:**
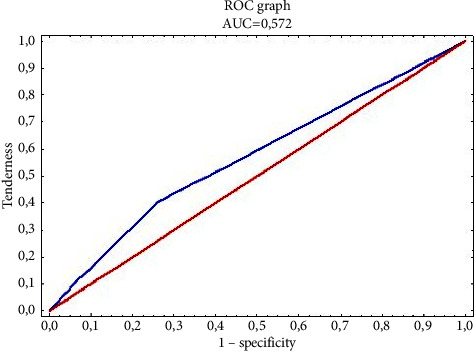
The plot of the ROC curve for the logistic regression model is moderately matched to the sources, i.e., specialist medical literature and specialist courses affecting the level of knowledge of the surveyed nurses in the field of nosocomial infections.

The value of the Hosmer–Lemeshow statistic is 1,172, with a *p* value = 0.556, which proves a significant fit of the logistic regression model. Based on the analysis of the area under the ROC curve, the model is also moderately fit to the data (area is AUC = 0.572) (Figure 4) and has a moderate predictive power, resulting from the obtained plots of sensitivity and specificity for different levels of probability.

### 3.3. Time since the Last Training and the Level of Knowledge among the Respondents

Participation in training is an important factor influencing the level of knowledge in the field of nosocomial infections. Searching for a relationship between the time that has elapsed since the last training and the state of knowledge in the field of nosocomial infections in the respondents, the variable defining the condition in question was coded in the form of ranks. The highest rank of “5” was defined as the longest time since the last training—three years and more, “4” more than two years ago, “3” two years ago, “2” a year since the last training, and “1” was assigned for the shortest time—in current year.

Using correlation analysis (Spearman's rho), a relationship was sought between time and general knowledge and its individual areas (raw numerical index) regarding hospital infections. A statistically significant relationship was found: the higher the index of general knowledge (*r* = −0.080; *p*=0.044) and knowledge for area I (*r* = −0.081; *p*=0.041), the shorter the time since the last training. The subjective assessment of the respondents' own knowledge also increases (*r* = −0.183; *p*=0.001) (Table 5).

### 3.4. Areas of Knowledge Affecting the Increase of Competences among the Respondents

Raising professional competence is an important factor influencing the level of knowledge about nosocomial infections. New technologies and solutions force the need for continuous improvement. The respondents indicated the areas of training and courses covering issues in terms of the possibility of infections in hospital wards, including: hand hygiene, use of personal protective equipment; disinfection and sterilization; postexposure procedure; preventing nosocomial infections by properly performing nursing procedures, i.e., caring for a patient with a urinary catheter, central catheter, changing dressings, toileting a bedridden patient; ways to monitor nosocomial infections; development of epidemic outbreaks, and the principles of rational antibiotic therapy.

The need to increase competence in the field of nosocomial infections was determined using logistic regression predictors. The dependent variable in the model was the willingness to participate in specialist courses and was defined as a dichotomous variable with two variants: yes (1) and no (0). The construction of the model was based on forward step regression, and the significance of the difference between successive, sequentially built models was assessed using the LR test (likelihood ratio).

The scope of content affecting the increase in competences has been presented in the form of a model in the table below (Table 6).

Based on the estimated logistic regression, it can be concluded that people declaring the improvement of their knowledge on nosocomial infections in the form of specialist courses will more often indicate the need to improve qualifications in the area of postexposure management (OR = 3,245; 95% CI: 2,096–5,025; *p*=0.001) and monitoring nosocomial infections (OR = 1,906; 95% CI: 1,215–2,990; *p*=0.005) compared to those who did not indicate this form of improvement (Table 6).

**Figure 5:**
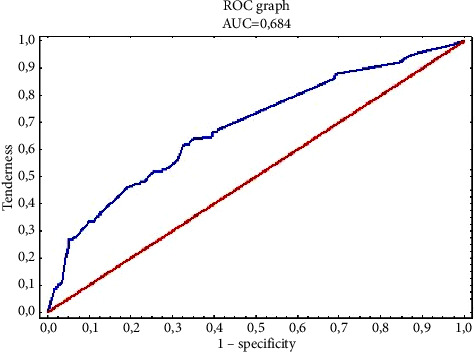
The plot of the ROC curve for the logistic regression model is moderately fitted to the predictors, i.e., postexposure management and methods of monitoring nosocomial infections, affecting the level of knowledge of the surveyed nurses in the field of nosocomial infections.

The value of the Hosmer–Lemeshow statistic is 5,750, with the value *p*=0.452, which indicates a significant model of fit of the logistic regression model. Based on the analysis of the area under the ROC curve, it can also be concluded that the model is well fitted to the data (area area is AUC = 0.684) (Figure 5) and has good predictive power resulting from the obtained sensitivity and specificity plots for different levels of probability.

### 3.5. General State of Knowledge of the Respondents in the Field of Nosocomial Infections

By checking the state of current knowledge of the nursing staff on nosocomial infections, three thematic areas were defined: the basic concepts of nosocomial infections; the microbiology of infections, including issues related to microbiological diagnostics; and ways to prevent nosocomial infections.

The knowledge of the nursing staff regarding nosocomial infections was checked using a standardized index. The general level of knowledge did not turn out to be statistically significantly differentiated by the type of ward (*p*=0.993). However, it should be noted that an insufficient level of knowledge was found in every third respondent from the conservative and surgical ward (97; 34.04% vs 119; 34.00%). In the case of area, I concerning basic concepts in the field of nosocomial infections, a statistically significantly higher level of knowledge was found in people working in medical wards (*p*=0.031). The level of knowledge for area II, microbiology of infections, including issues related to microbiological diagnostics (*p*=0.069) and for area III, methods of preventing nosocomial infections (*p*=0.625), turned out to be at a similar level, where only every second person surveyed was at the level of sufficient. Existing deficiencies in the professional competences of nurses and male nurses were found (Table 7).

The first area covered the basic concepts of nosocomial infections, which consisted of 6 statements covering the following issues: definition of nosocomial infection, system of preventing and combating nosocomial infections, clinical forms of nosocomial infections, definition of endo- and exogenous infections, antibiotic therapy, and selected ways of preventing nosocomial infections (procedures after-exposure).

Statistically significantly more often (*p*=0.001) the correct definition of nosocomial infection was indicated by the respondents from medical wards (228; 80.00% vs 240; 68.57%). They defined nosocomial infection as any infection that was not in the incubation phase at the time of admission to the hospital, and symptoms occurred 48–72 hours after admission to the hospital or after discharge from the hospital within a period not longer than the longest incubation period. Every fifth person (57; 20.00%) working in conservative wards and every third (110; 31.43%) in surgical wards incorrectly defined nosocomial infection, defining it most often as any infection that was found during the patient's hospital stay and was caused by multidrug-resistant microorganisms present in the hospital environment.

Statistically significantly more often (*p*=0.001) the respondents from medical wards (278; 97.54% vs 315; 90.00%) correctly defined the procedure for implementing and ensuring the functioning of the system for preventing and combating nosocomial infections as mandatory for all hospitals, imposed by law. The most frequently selected incorrect answer was the statement that the procedure in question is obligatory for clinical, specialist, and provincial hospitals, and voluntary for powiat hospitals.

Every second person, both working in conservative and surgical wards (*p*=0.206), was able to correctly determine that the most common form of clinical nosocomial infection is urinary tract infection (152; 53.33% vs 169; 48.29%). The incorrect answer indicated by the subjects in both groups was that it is pneumonia. It was found that the respondents did not have sufficient competence (knowledge) in the discussed area.

The correct definition that endogenous infections are caused by microorganisms from the patient's own physiological flora (natural microbiota) was more often indicated by the respondents working in conservative wards than in surgical wards (207; 72.63% vs 231; 66.00%), but this difference turned out to be statistically insignificant (*p*=0.067). Every third respondent incorrectly indicated the source of these infections, pointing to the environment of a given hospital/patient's surroundings.

The next area covered knowledge in the field of antibiotic therapy and the determination of therapy, which consists in selecting the drug in accordance with the identification of the pathogen and determining its drug susceptibility. The vast majority of respondents answered the question correctly, pointing to targeted therapy (269; 94.39% vs 326; 93.14%, *p*=0.521). Combination therapy was the incorrectly chosen answer.

According to the respondents, the potential source of HBV, HVC and HIV infection is blood and any biological material containing blood and semen, pre-ejaculate, and vaginal discharge. This is the correct answer, which was indicated equally often (*p*=0.383) by the respondents from the conservative and surgical wards (251; 88.07% vs 300; 85.71%). Incorrect answers included statements that only blood and any biological material containing blood are such a source (Table 8).

The second area (6 items) focused on the microbiology/infections, taking into account issues in the field of microbiological diagnostics and etiological factors of nosocomial infections.

The respondents indicated *Staphylococcus aureus*, *Escherichia coli*, and *Pseudomonas aeruginosa* as the most common etiological factor of nosocomial infections for the entire hospital, where statistically significantly more often (*p*=0.048) correct indications were found in people working in medical wards (218; 76.49% vs 243; 69.43%). The most frequently indicated incorrect answer was that this agent is *Streptococcus pyogenes*, *Klebsiella pneumoniae*, and *Acinetobacter baumannii*.

According to the respondents, the microorganism that most often contributes to the occurrence of hospital diarrhea in adults is *Clostridioides difficile*. The frequency of correct indications in both study groups (247; 86.67% vs. 291; 83.14%) was statistically insignificantly differentiated (*p*=0.220). The most common incorrect answer in the discussed area was the indication of *Escherichia coli* EPEC (enteropathogenic strains of *Escherichia coli*).

According to the respondents, the most common ways of transmission of Staphylococcus aureus in the hospital environment are the hands of medical personnel (202; 70.88% vs 236; 67.43%, *p*=0.350). Unfortunately, every third respondent could not correctly indicate the routes of its transmission, pointing to surgical tools. The hands of nursing staff play an important role in the transmission of microorganisms and thus contribute to the spread of nosocomial infections.

If sepsis is suspected in a patient with a central venous line, blood should be collected from the central line and 2 separate peripheral lines for microbiological testing. This procedure was indicated only by every second respondent (131; 45.96% vs 172; 49.14%, *p*=0.425). The others pointed to an incorrect operation taking blood from a central venous line and one peripheral venous line. This allows for the conclusion that knowledge in the discussed area is insufficient.

The time after which the result of a microbiological urine test with an antibiogram is obtained is 2-3 days. Statistically significantly more often (*p*=0.032) the correct answer was indicated by the staff from conservative wards (206; 72.28% vs 225; 64.29%). The most common incorrect answer was the term waiting time after one week.

The last element in the field of microbiology of infections and microbiological diagnostics covered strains that are classified as alarm factors. The vast majority of respondents correctly identified methicillin-resistant *Staphylococcus aureus* (MRSA), vancomycin-resistant *Enterococcus faecium* (VRE), and *Escherichia coli* producing ESBL beta-lactamase (223; 78.25% vs 265; 75.71%, *p*=0.452). The erroneous indication was to select only one strain: methicillin-resistant *Staphylococcus aureus* (MRSA) (Table 9).

The third area, i.e., the last one, covered various ways to prevent nosocomial infections and contained 8 questions.

In the case of the question regarding perioperative antibiotic prophylaxis, the correct answer is that it is a short (usually one dose) administration of an antibiotic (usually cefazolin) just before the procedure in a contaminated clean field or a clean field with a high risk of infection. This was statistically significantly more often (*p*=0.028) given by nurses and nurses working in surgical wards (99; 28.29% vs 59; 20.70%). Every 7th respondent incorrectly indicated the answer as a short (usually one dose) administration of an antibiotic (usually cefazolin) just before each surgical procedure.

According to the respondents, the correct management of a patient in whom the *Klebsiella pneumoniae* NDM strain was detected in a rectal swab taken at admission to the hospital as part of screening tests is to isolate the patient until the end of the hospital stay in a separate room or cohort with patients who have been diagnosed with the same microorganism; therefore, antibiotic therapy should not be implemented. The frequency of correct indications in both study groups (106; 37.19% vs 108; 30.88%) was statistically insignificantly differentiated (*p*=0.093). The most frequently incorrectly indicated answer was treatment of the patient with an antibiotic in accordance with the antibiogram and isolation or cohort until the end of antibiotic therapy. Appropriate management of a patient diagnosed with the *Klebsiella pneumoniae* NDM strain is important due to the fact that this bacterium is resistant to most available antibiotics and spreads very easily in the hospital environment.

With the exception of contact with patients infected with *Clostridioides difficile*, the recommended method of hand hygiene during nursing procedures is hand disinfection using an alcohol-based hand rub. The vast majority of respondents correctly indicated this answer (207; 72.63% vs 256; 73.14%). The most frequently and incorrectly chosen answer was that if the hands are visually clean, then only hand disinfection using an alcohol-based agent, and washing and subsequent disinfection only when the hands are dirty.

When asked when nonsterile disposable gloves should be used, the correct answer was that during contact with body fluids, excretions, and secretions of the patient and during all activities with an isolated patient who is colonized/infected with an alert microorganism, this (*p*=0.376) was indicated by the subjects from the conservative and surgical wards (232; 81.40% vs 275; 78.57%). Incorrectly, the most common response was to use nonsterile disposable gloves only when in contact with body fluids, excretions, and secretions of the patient.

Statistically significantly more often (*p*=0.014) the respondents from the medical wards (176; 61.75% vs 182; 52.00%) correctly identified the personal protective equipment that should be provided to the nursing staff performing activities with a patient covered by air-dust isolation (gloves, protective apron, filtering half-mask). The most frequently selected incorrect answer was gloves, a protective gown, and surgical mask.

Containers intended for medical waste with sharp ends or edges should be replaced when filled to a maximum of 2/3 of the volume, but at least once every 2-3 days. This answer was correctly indicated by the majority of respondents (235; 82.48% vs 287; 82.00%, *p*=0.881). The incorrectly selected answer was to replace the containers after filling up to 2/3 of their volume, regardless of the time of their use.

When asked how long an alcohol agent should remain on the skin in order to properly disinfect the patient's skin before taking blood for laboratory tests, the nursing staff correctly answered that until it dries or according to the manufacturer's information on the preparation's packaging (228; 80.00% vs 292; 83.43%, *p*=0.264). The most frequently incorrectly indicated answer was about 10 seconds.

The last question in this area concerned the correct procedure after a needle stick that had previously been used for intravenous injection. The skin should be washed with plenty of lukewarm water and soap, do not squeeze the wound; do not stop bleeding; do not use alcohol-based disinfectants immediately after a cut or puncture; put on a sterile dressing; and report the case of occupational exposure in the workplace. This answer was indicated as correct by the majority of respondents in both groups (267; 93.68% vs. 317; 90.57%). The most frequently incorrectly indicated answer was to stop bleeding immediately after a cut or needle stick, not to use alcohol-based disinfectants, to apply a sterile dressing, and to report a case of occupational exposure in the workplace (Table 10).

## 4. Discussion

Healthcare-associated infections are a global problem and a major threat to the safety, health, and lives of patients [20]. Reducing the number of nosocomial infections is possible thanks to rigorous adherence to medical procedures as well as consolidating and updating the knowledge of medical staff in the fields of epidemiology, etiology, transmission routes, and, above all, methods of infection prevention. One of the most effective procedures to prevent the spread of infection, both in hospitals and in everyday life is hand hygiene [21]. However, despite the fact that it is a simple procedure, the theoretical and practical knowledge among medical personnel, including nursing staff, is not always satisfactory. Therefore, topics related to hand hygiene should be a permanent part of health education, not only for medical personnel but also for patients [22]. The importance of education as an important element of infection prevention is emphasized in many studies. The problem should be approached multidimensionally, and the increasing frequency of infections among hospitalized patients must be accompanied by increasing the awareness of medical staff and continuous training in this area [23]. The obtained research results can be easily transferred to the entire region of Poland due to the fact that Kielce is the capital of the Świętokrzyskie Voivodeship, where there are representative hospitals from every level of reference, which can undoubtedly be applied to other centers such as Łódź or Warsaw.

### 4.1. Sociodemographic Factors

The study showed that the level of knowledge about nosocomial infections is influenced by the education, specialization, and age of the surveyed nurses. Similar results were obtained by the authors of a study conducted in Zhejiang Province, China, in which doctors and nurses working in neonatal intensive care units took part, and the subject of the study was their knowledge and attitudes in the field of prevention and control of nosocomial infections caused by multidrug-resistant organisms. The knowledge of the surveyed people was correlated with gender, education, referral status of the hospital, and additionally with regular supervision and training [24]. A study conducted among nurses in India also showed a statistically significant relationship between infection control knowledge, seniority, and the place of work of nurses [25]. On the other hand, Turkish research, which assessed the impact of various factors on the level of knowledge in the field of hand hygiene before and after the training, did not show a statistically significant effect of seniority on the knowledge of this issue but showed a statistically significant relationship between the increase in knowledge after the training and variables such as marital status, gender, and type of ward [26]. In an interesting study conducted among nonmedical staff from various hospitals in Iran, a statistically significant correlation was obtained between seniority, type of hospital, and knowledge and attitudes regarding the control of nosocomial infections [27]. The work of authors from Uganda, evaluating the knowledge of representatives of various medical professions about the resistance of microorganisms to antibiotics and rational antibiotic therapy, revealed statistically significant differences between knowledge and practice. Nurses had lower knowledge compared to doctors and pharmacists [28]. Another study from India provided knowledge that statistically significant elements that influenced the state of knowledge among nursing staff about the so-called universal precautions are as follows: gender, place of residence, and education [29]. The impact of similar factors (gender, age, and type of employment) on the knowledge of health professionals in the field of healthcare-associated infections was shown by subsequent studies by Chinese authors. In addition, it was shown that people who participated in clinical consultations with infectious disease doctors had greater knowledge [30]. A study in the United Kingdom assessing knowledge of recommendations for the prevention of methicillin-resistant Staphylococcus aureus (MRSA) infection showed the influence of medical specialty on knowledge in this area. In the knowledge-testing survey, the best results were obtained by people specializing in anesthesiology and intensive care, while the weakest people with diagnostic specializations, i.e., radiology, biochemistry, or laboratory medicine. In addition, it was found that the results of epidemiological nurses [8, 31] were higher than those of physicians (8.69 and 6.6 points out of 1, respectively) [32]. Researchers from Ethiopia have shown that although education and professional experience have an impact on medical workers' theoretical knowledge in the field of infections, this knowledge does not translate into appropriate practice. Continuous on- and off-work training and continuous updating of medical procedures related to infection prevention can fill this gap [19].

### 4.2. Sources of Knowledge

The sources of knowledge that had an impact on the sufficient level of knowledge among the respondents were specialist medical literature and specialist courses, and the most frequently indicated source of knowledge were internal trainings conducted in the workplace. In Italy, the sources of knowledge indicated by the respondents in the prevention of SSI were guidelines in this area (73.60%) and similar training courses (51.60%) [33]. In Pakistan, the nursing staff mainly gained knowledge from doctors (72.30%), and the other sources of knowledge were training and the Internet (6.90%) [34].

### 4.3. Time since Last Training

A statistically significant relationship was also found: the shorter the time since the last training, the higher the level of knowledge among nursing staff. Researchers from other countries have reached the same conclusions. In Turkey, nurses who received training in hygienic hand washing increased their knowledge in this area. All nurses had a statistically significantly higher level of knowledge than before the training, and the percentage of correct answers exceeded 90.00% [26]. A study conducted in India on the knowledge, attitudes, and practices of nursing staff in the field of infection control also showed that after the training intervention, knowledge among staff increased from 9.42 to 12.98 one week after the training, and after a month, it was still high and amounted to 12.18. In addition, it resulted in a reduction in the incidence of urinary tract infections and intravascular catheter-related infections [35]. The same data were provided by a study in Switzerland, where knowledge after a training intervention increased, especially among nurses and medical staff who did not perform managerial functions [36]. In Spain, nurses' knowledge of venous line recommendations also improved with 4 out of 14 instructions following a training program [37]. In a comparative study among Ethiopian and Chinese nurses, more Chinese nurses received training (54.40%) compared to Ethiopian nurses (41.70%). Among Chinese nurses, the vast majority reported regular supervision, monitoring, and monthly training on the prevention of nosocomial infections compared to Ethiopia, where hospitals conducted regular educational programs, but only for new employees [38]. A study in Poland provided other conclusions. The time that has passed since the last training did not significantly affect the level of knowledge, which was at a comparable level among the surveyed nursing staff, while participation in training on nosocomial infections resulted in a higher level of knowledge among the respondents [39]. Kong et al., in their study, checked the knowledge of medical workers about nosocomial infections and hand hygiene in endoscopy rooms before and after the introduction of the PDCA method, which is a modus operandi of continuous improvement. The results confirmed that the knowledge of the surveyed people was statistically significantly higher than in the control group [40]. To sum up, it can be noted that participation in training is a very important factor influencing the level of knowledge among medical staff.

### 4.4. Areas of Knowledge

In this study, nursing staff had the highest knowledge of basic concepts regarding nosocomial infections (59.21%). Similarly, in Italy, the area in which the level of knowledge was the highest was general issues regarding nosocomial infections [41], while in Iran, most respondents (90.90%) demonstrated the highest knowledge in the field of hand hygiene and medical waste management [27]. Similarly, in the United States, the highest knowledge among nurses was reported in issues related to hand hygiene and handling sharps instruments [42].

### 4.5. State of Knowledge

Many researchers point out that properly educated and trained nursing staff who follow all prevention rules, such as hand hygiene or isolation, significantly reduce hospital infections. Many studies have also confirmed that it is impossible to replace qualified nursing staff. In intensive care units, when there is an insufficient number of nursing staff or when nurses do not have sufficient knowledge about the prevention and control of nosocomial infections, this significantly increases the incidence of nosocomial infections, complications, and patient deaths. Index SENIC (Study of the Efficacy of Nosocomial Infection Control) found that hospitals reduced nosocomial infections by approximately 32% when their infection surveillance and control program included four components, two of which involved skilled and knowledgeable staff: at least one full-time infection control specialist for every 250 beds and a trained hospital epidemiologist [43]. Other researchers have also proven the relationship between education and knowledge and the occurrence of nosocomial infections. Urinary tract infections decreased by 9% among patients cared for by nurses with higher education and, therefore, greater knowledge; the same correlation occurred in patients with pneumonia, where this percentage decreased by 6%. Other research conducted by Cho et al. also confirmed this correlation that skilled nurses reducing patients' incidence of pneumonia by 10% [44]. Also, Needleman et al. came to the same conclusions. The association between qualified staff was associated with a shorter hospital stay and a lower incidence of nosocomial urinary tract infections and upper gastrointestinal bleeding. In addition, urinary tract infections decreased among surgical patients [45]. Olatade et al. found a statistically significant relationship between knowledge and their preventive practice against nosocomial infections among health care workers, which is consistent with appropriate expectations [46].

In the study, the general knowledge of nurses and male nurses regarding nosocomial infections was at a sufficient level for the majority of respondents (65.98%). Other data were provided by a Polish study conducted among nursing staff, whose level of knowledge on postexposure prophylaxis and contact-transmitted infections was insufficient [39]. Meanwhile, a sufficient level of knowledge has also been obtained by scientists in other countries. In India, the knowledge of hand hygiene among medical staff was 66.4% ± 27.5% [47] and the awareness of nursing staff was 69.25%. The general state of knowledge about nosocomial infections was at a very good level (above 70.00%) [29]. In Pakistan, 65.56% of respondents had adequate knowledge of nosocomial infections [23], while in another study also conducted in the same country, the average result of knowledge was higher and amounted to 79.94 ± 20.67, and 56.00% of nursing staff had good knowledge [34]. High results were also obtained by researchers in Italy, checking the knowledge of nurses in the prevention and control of nosocomial infections. Among the respondents, 75.80% of people had a sufficient level of knowledge [48]. A similar level of knowledge was obtained by nonmedical staff working in hospitals in Iran in the field of nosocomial infection control. The vast majority of staff (75.00%) had adequate knowledge, and the mean score was 11.2 ± 2.2 (range: 3–15) [27]. Very high results were obtained in the study by Tash et al. where the level of knowledge about hand hygiene practices and the use of personal protective equipment among nurses was as high as 94.40% (85/90) [49]. Nurses in Kosovo had similarly high knowledge; the general level of knowledge on the spread of nosocomial infections was 90.00% [50]. In the United States, nurses also had a good knowledge of nosocomial infection control [42]. In Nepal, however, the level of knowledge was much lower; only 57.10% of the nursing staff had the appropriate knowledge, and the average of the ratings was 27.75 out of 38 [51]. Equally low knowledge was found in African countries, i.e., Ethiopia, where only 45.50% of nurses had sufficiently good knowledge on the prevention of nosocomial infections [52]. A similar study in Cyprus showed that nurses do not have sufficient knowledge about nosocomial infections in geriatric patients [53]. The same data was provided by another study conducted in Poland. The nursing staff did not have sufficient knowledge about urinary tract infections. The highest scores were obtained by respondents with basic knowledge of urinary tract infections [54]. In Bulgaria, knowledge about the prevention and control of nosocomial infections exists among healthcare professionals but is at a very basic level. Nurses derive their knowledge from their daily work in the hospital [55]. In Australia, the knowledge of nurses has also not reached a high level. The median assessment of ICU staff knowledge of VAP was 6/10 (IQR: 5–7) [56]. In South Korea, the level of hand hygiene knowledge among nursing staff was also low [57]. A low level of knowledge about CRBSI was also found among Jordanian ICU nurses. The average knowledge score was 3.3, and SD was 1.8 (out of 10) [58].

The study showed that the level of knowledge is statistically significantly different and higher in the group of staff working in conservative wards compared to surgical wards, and the median in both groups was 15. Different results were obtained in a study in Italy among nurses working in surgical wards. The nursing staff in surgical wards had a higher level of sufficient knowledge (78.60%) compared to nurses from other wards (73.60%) [48]. Another study conducted in Italy found a similar relationship. Knowledge among the respondents was higher in the people who work in intensive care units [59]. A study in Pakistan also showed that people working in gynecology and surgery, i.e., surgical departments, had higher knowledge than people working in other departments [34]. In Poland, however, the type of ward did not have a statistically significant effect on the general level of knowledge of medical personnel about contact-transmitted infections [60].

In our study, when asked about the correct duration of skin disinfection in a patient before collecting blood for laboratory tests, the respondents correctly indicated that the alcoholic agent should dry or in accordance with the manufacturer's recommendations on the preparation's packaging (81.89%). In Pakistan, the correct duration of hand washing was indicated by fewer staff (73.70%) [61]. A similar study was conducted in Germany, assessing the knowledge and behavior of nurses and nursing managers regarding hand hygiene in nursing homes. The respondents obtained comparable results (79.00%) in the field of effective hand hygiene methods, answering that 30 seconds is the correct time for hand disinfection [62]. In India, significantly fewer people (54.00%) [47] answered the same question correctly. In another study in India, the respondents' knowledge about the appropriate duration of hygienic and surgical hand washing and the sequence of removing personal protective equipment was at a similar level and amounted to 60.00–70.00% [29].

In our study, nursing staff, when asked about the choice of the recommended method of hand decontamination while performing nursing procedures, except for contacts with patients infected with *Clostridioides difficile*, respondents correctly indicated that it was hand disinfection using an alcohol-based agent (72.91%). Yanke et al. in their study focusing on the prevention of *Clostridioides difficile* infections indicated that some employees wrongly believed that alcohol-based hand sanitizer was an effective method of decontaminating hands after leaving the room of a patient infected with this anaerobic bacterium [63]. In Italy, much fewer respondents (28.50%) knew that washing hands with soap and water was the right way to prevent the spread of nosocomial infections with this bacterium [64].

In our study, when asked what most often causes hospital staphylococcal infection, nursing staff indicated that it is in the hands of medical staff (68.98%). In India, when asked about the main route of spread of microorganisms among patients in health facilities, more respondents (88.00%) answered correctly that it is the contaminated hands of medical staff and indicated the hospital environment as the most common source of pathogens responsible for infections associated with the provision of health services (40.9%) [47]. Nurses working in Kuwait (73.60%) [65] and Saudi Arabia (77.80%) also showed higher knowledge in this area, indicating the same answer [66]. Nurses working in Kuwait (68.50%) were able to identify 5 moments of hand hygiene [65], and medical students in Slovakia (67.10%) had sufficient knowledge of observing hand hygiene rules [21]. An interesting study was also conducted among medical and nursing students in Greece, where future nurses (60.40%) had higher knowledge than future doctors (57.20%) about hand hygiene [67]. In Greece, during qualitative interviews, medical staff admitted that they did not have sufficient knowledge, especially regarding hand hygiene, wrongly believing that its main purpose was solely to protect staff [68]. Among Dutch nursing staff, the majority of people stated that they had knowledge of “how to work hygienically” and how to explain to patients the purpose of preventing and controlling nosocomial infections [69]. In the United States, only 45.40% of nurses indicated that they always wash their hands before inserting a urinary catheter [31]. In Italy, the vast majority of respondents (91.00%) believed that they always performed hand antiseptics, both before and after invasive procedures, such as catheterization of the urinary bladder or insertion of a cannula into a peripheral vein [33].

When asking the respondents when to use nonsterile disposable gloves, they most often answered that during contact with the patient's body fluids, excretions, and secretions and when performing all activities with an isolated patient who is colonized with an alarm factor (79.84%). A study in India on hand hygiene knowledge, attitudes, and practices found that respondents were less knowledgeable. The study showed that 59.00% of staff believed that wearing gloves could replace hand washing or disinfection [70]. The same view was held among Dutch nurses in a study conducted by Lescure et al. [71]. In the United States, health care workers interviewed reported that they always wear gloves as part of standard precautions. In addition, most employees declared that they use gloves in situations where they are not necessary and always perform hand hygiene before putting on gloves [72].

In our study, the majority of nursing staff (91.97%) knew the correct procedure for following a needle stick injury that had previously been administered intravenously. Medical staff in Nepal showed higher knowledge (98.80%), indicating that large amounts of lukewarm water and soap should be used after an incidental injury with a sharp medical instrument [51]. In turn, staff in India had lower knowledge in this area, where 78.00% of respondents [47] and 50.00% of nurses knew what to do in the event of a needle stick injury [73].

When examining the knowledge of nursing staff regarding the time after which sharp-edged medical waste containers should be replaced, our respondents answered that after they were filled to a maximum of 2/3 of their volume, but at least once every 2-3 days (82.20%). Respondents in India showed lower knowledge (70.00%) in the proper management of acute medical waste [29], as well as in Nepal, where only half of the respondents believed that an appropriate system and supervision of medical waste are factors influencing the prevention of infections hospital [51]. In Italy, however, nurses indicated that 21.00% of containers intended for medical waste were filled to more than ¾ of their capacity, both in surgical and medical wards [48].

When asked about the potential sources of HBV, HCV, and HIV infection, our respondents answered that it was blood and any biological material containing blood, as well as semen, pre-ejaculate, and vaginal secretions (86.77%). Health care workers in Georgia [74] had an insufficient level of knowledge regarding the above issue, and in Nigeria, the general knowledge of the subject and postexposure procedures among respondents was very low [75].

When asked about the personal protective equipment that medical personnel should be equipped with in the case of air-dust isolation, our respondents answered that they should have gloves, a protective apron, and a filtering half-mask (56.38%). In Spain, more nurses (90.00%) knew that they should wear a mask and protective glasses when suctioning secretions from the trachea [76]. In Nepal, most nurses reported wearing gloves, a mask, and goggles to protect themselves from blood and body fluids [51]. In Jordan, most respondents had knowledge about isolation precautions, but questions about air and contact isolation were the most difficult, and fewer respondents knew the correct answer (40.70%) [77]. In Nigeria, only 8.00% of staff were able to name the types of isolation, and 17.80% believed that personal protective equipment was necessary for isolation. Also, a small number of people (14.50%) were able to name situations in which such isolation is necessary [78]. In Italy, the vast majority of respondents (93.20%) claimed to use disposable protective equipment on patients with infectious diseases [33].

When asked about the use of antibiotics based on the antibiogram, respondents indicated that it was a targeted therapy (93.70%). A similar study was conducted in the United States, where fewer nurses (64.20%) were familiar with the terms antibiotics and antimicrobial stewardship [79], and in the study by Greendyke et al., a similar number of staff (62.00%) did not have sufficient knowledge [80]. The issue of antibiotic resistance also caused great difficulty among respondents in Saudi Arabia; only 29.10% of nurses knew the correct definition [81]. In Singapore, as many as 38.60% of nurses admitted that they had no or limited knowledge of antimicrobial management [82].

The nursing staff had a good knowledge of alarm microorganisms that are resistant to one or more classes of antibiotics (76.85%). In the Netherlands, fewer nurses felt they had sufficient knowledge about MDROs and were able to provide answers to patients in this area. The average knowledge score was 4.5 (range 0–10), and 2/3 of the respondents would like to increase their knowledge on this topic [69]. In Greece, among the nursing staff working in the ICU, during the interview, few respondents reported information about infections that develop there, and no nurse mentioned the colonization and spread of MDROs [83].

Nurses had a big problem with giving the correct answer regarding perioperative antibiotic prophylaxis. Only 24.88% of respondents knew that one dose of cefazolin should be administered just before the procedure in a clean contaminated field or in a clean field with a high risk of infection. In Italy, even fewer staff (14.10%) knew the correct duration of antibiotic prophylaxis (<24 hours after surgery) in their department [33].

### 4.6. Limitations of the Study

A limitation of the study in terms of assessing the differences between the sexes may be the small number of men (only 6%) among the respondents. This is due to the fact that the vast majority of nurses are women. The study may also be limited by the time interval between the beginning of the study and its completion, reflecting the time between the distribution of the questionnaires and their return by the respondents. This may raise doubts as to the independence of the answers provided by the respondents. This fact could affect the credibility of the obtained results. Another weakness of the study is the lack of a test validation of the instrument; only the Cronbach's alpha coefficient was used, which indicated that the tested questionnaire is a reliable and a valid tool. The analysis confirmed its reliability in terms of basic psychometric properties. Reliability is demonstrated by high values of Cronbach's alpha coefficient. All questions and statements included in the questionnaires were understandable to the respondents. As a process, validation involves collecting and analyzing data to assess the accuracy of an instrument. There are numerous statistical tests and measures to assess the validity of quantitative instruments, which generally involve pilot testing. Another limitation is the lack of a pilot test of the instrument which should be carried out on a small number of people in order to verify whether the selected methods, techniques, and tools are appropriate for the studied group.

## 5. Implications for Nursing Management

Nosocomial infections are an integral part of the provision of health services, so it is important to know how to minimize the risk of infections. Particular attention should be paid to the continuous, frequent, and regular training of nurses in the field of nosocomial infections, because the knowledge of nursing staff is insufficient. The acquired specialized knowledge must be translated into the everyday preventive behaviors of nurses and also try to supervise and verify nurses' behavior. Knowledge must be followed by specific preventive behaviors. It is advisable to develop procedures and research tools in the health care system that would be helpful both in assessing and acquiring knowledge about nosocomial infections.

## 6. Conclusions

The level of knowledge about nosocomial infections among nursing staff is insufficient. Knowledge is diverse, and its main determinants are specialization, education and age. A sufficient level of knowledge among the respondents is conditioned primarily by the use of specialist literature and participation in specialist courses, which determine both the scope and area of knowledge about nosocomial infections. Nursing staff working in conservative wards are characterized by a higher level of knowledge in the area of basic concepts related to nosocomial infections. The area of knowledge that will be strengthened is the microbiology of infections, including issues related to microbiological diagnostics. Postexposure management and methods of monitoring nosocomial infections are the areas in which the respondents most often would like to deepen their knowledge.

## Figures and Tables

**Table 1:** Detailed characteristics of the study group of nurses.

Sociodemographic data		*n*	%
Sex	Woman	597	94.00
	Man	38	6.00

Age	Up to 30 years	205	32.30
	From 31 to 39 years	138	21.70
	From 40 to 49 years	142	22.40
	Over 50 years old	150	23.60

Education	Medical highschool	49	7.70
	Medical professional study	49	7.70
	Higher professional (bachelor of nursing)	166	26.10
	Higher master's nursing	371	58.40

Specialization	Yes	255	40.00
	No	380	60.00

Seniority	Up to 10 years	276	43.50
	From 10 to 20 years	126	19.80
	Over 20 years old	233	36.70

Branch type	Conservative	285	45.00
	Treatment	350	55.00

**Table 2:** The level of knowledge of the respondents (raw numerical values) due to selected sociodemographic factors and the type of ward as a place of work.

Variables	*n*	Me	Q1	Q3	Min	Max	*p* value
Generally	635	15	3	20	13	16	—

Woman	597	15	13	16	3	20	0.437^A^
Man	38	14	12	16	5	18	

Conservative	285	15	13	17	6	20	0.016^A^^*∗*^
Treatment	350	15	12	16	3	19	

No specialization	380	14	12	16	3	19	0.001^A^^*∗*^
Having a specialization	255	15	13	17	4	20	

Age up to 30 years	205	14	13	16	5	20	0.026^B^^*∗*^
Age from 31 to 39 years	138	15	13	16	4	19	
Age from 40 to 49 years	142	15	13	17	4	19	
Age over 50	150	14	12	16	3	20	

Medical highschool	49	13	11	15	4	18	0.004^B^^*∗*^
Medical professional study	49	14	12	16	8	19	
Higher professional (bachelor of nursing)	166	14	12	16	3	19	
Higher Master's nursing	371	15	13	16	4	20	

Work experience -up to 10 years	276	15	13	16	5	20	0.434^B^
Work experience -from 10 to 20 years	126	15	13	16	3	19	
Work experience -over 20 years	233	14	12	17	4	20	

^A^Mann–Whitney *U* test, ^B^Kruskal–Wallis rank ANOVA test, ^*∗*^*p* < *α*, *α* = 0.05.

**Table 3:** Predictors affecting the level of knowledge of the surveyed nurses in the field of nosocomial infections.

Variable -reference variant	Estimation of the logistic regression parameter	OR (95% CI)	*p* value
Free expression	0.717	2.048 (1.568–2.676)	0.001
Education: medical high school	−0.888	0.415 (0.294–0.998)	0.004
Education: medical vocational studies	−0.613	0.532 (0.294–0.998)	0.049
Education: higher professional (bachelor of nursing)	−0.308	0.735 (0.497–1.087)	0.123
Having a specialization	0.398	1.489 (1.050–2.112)	0.026

**Table 4:** Sources affecting the level of knowledge of the surveyed nurses in the field of nosocomial infections.

Variable -reference variant	Estimation of the logistic regression parameter	OR (95% Cl)	*p* value
Free expression	0.472	1.603 (1.320–1.948)	0.001
Specialized medical literature	0.477	1.612 (1.068–2.432)	0.023
Specialized courses	0.478	1.613 (1.007–2.581)	0.047

**Table 5:** Relationship between the time since the last training and particular dimensions of knowledge about nosocomial infections.

Time since last training a:	Spearman's rho	*t*(*N* − 2)	*p* value
Raw indicator of general knowledge	−0.080	−2.017	0.044^*∗*^
Raw knowledge index: area I	−0.081	−2.049	0.041^*∗*^
Raw knowledge index: area II	−0.052	−1.316	0.188
Raw knowledge index: area III	−0.039	−0.977	0.329
Subjective assessment of the state of knowledge	−0.183	−4.673	0.001^*∗*^

^*∗*^ *p* < *α*; *α* = 0.05 a statistically significant relationship was found.

**Table 6:** Predictors affecting the level of knowledge of the surveyed nurses in the field of nosocomial infections.

Variable -reference variant	Estimation of the logistic regression parameter	OR (95% Cl)	*p* value
Free expression	−2.022	0.132 (0.099–0.177)	0.001
Postexposure procedures	1.177	3.245 (2.096–5.025)	0.001
Methods of monitoring nosocomial infections	0.645	1.906 (1.215–2.990)	0.005

**Table 7:** The general state of knowledge of the surveyed nurses in the field of nosocomial infections.

Area	Together (*n* = 635; %)	Conservation ward (*n* = 285; %)	Treatment department (*n* = 350; %)	*p* value^*∗*^
General knowledge about nosocomial infections				
Sufficient	419 (65.98)	188 (65.96)	231 (66.00)	0.993
Insufficient	216 (34.02)	97 (34.04)	119 (34.00)	
Area I: basic concepts of nosocomial infections				
Sufficient	376 (59.21)	182 (63.86)	194 (55.43)	0.031^*∗∗*^
Insufficient	259 (40.79)	103 (36.14)	156 (44.57)	
Area II: microbiology of infections, including issues related to microbiological diagnostics				
Sufficient	280 (44.09)	137 (48.07)	143 (40.86)	0.069
Insufficient	355 (55.91)	148 (51.93)	207 (59.14)	
Area III: ways to prevent nosocomial infections				
Sufficient	292 (45.98)	128 (44.91)	164 (46.86)	0.625
Insufficient	343 (54.02)	157 (55.09)	186 (53.14)	

The asterisks indicate as follows: ^∗^Test χ^2^, ^∗∗^*p* < *α*; *α* = 0.05 statistical significance was found.

**Table 8:** Area I: basic concepts of nosocomial infections.

Components of the area I/type of response	Together (*n* = 635; %)	Conservation ward (*n* = 285; %)	Treatment department (*n* = 350; %)	*p* value^*∗*^
A nosocomial infection is considered to be any infection that was not in the incubation phase at the time of admission to the hospital, and the symptoms appeared 48–72 hours after admission to the hospital or after discharge from the hospital within a period not longer than the longest incubation period				
Correct	468 (73.70)	228 (80.00)	240 (68.57)	0.001^*∗∗*^
Incorrect	167 (26.30)	57 (20.00)	110 (31.43)	
The implementation and operation of a system for preventing and combating nosocomial infections is mandatory for all hospitals, imposed by law				
Correct	593 (93.39)	278 (97.54)	315 (90.00)	0.001^*∗∗*^
Incorrect	42 (6.61)	7 (2.46)	35 (10.00)	
The most common clinical form of nosocomial infections is urinary tract infection				
Correct	321 (50.55)	152 (53.33)	169 (48.29)	0.206
Incorrect	314 (49.45)	133 (46.67)	181 (51.71)	
Endogenous infections are caused by microorganisms from the patient's own physiological flora (natural microbiota)				
Correct	438 (68.98)	207 (72.63)	231 (66.00)	0.072
Incorrect	197 (31.02)	78 (27.37)	119 (34.00)	
The use of an antibiotic selected on the basis of an antibiogram is a targeted therapy				
Correct	595 (93.70)	269 (94.39)	326 (93.14)	0.521
Incorrect	40 (6.30)	16 (5.61)	24 (6.86)	
A potential source of infection with HBV, HCV and HIV viruses is blood and any biological material containing blood and semen, pre-ejaculation, and vaginal secretion				
Correct	551 (86.77)	251 (88.07)	300 (85.71)	0.383
Incorrect	84 (13.23)	34 (11.93)	50 (14.29)	

^*∗*^Test *χ*^2^; ^*∗∗*^*p* < *α*; *α* = 0.05 statistical significance was found.

**Table 9:** Area II: microbiology of infections, including issues related to microbiological diagnostics.

Components of area II/type of response	Together (*n* = 635; %)	Conservation ward (*n* = 285; %)	Treatment department (*n* = 350; %)	*p* value^*∗*^
Microorganisms that are the most common etiological agents of nosocomial infections (generally for the entire hospital) are *Staphylococcus aureus, Escherichia coli, Pseudomonas aeruginosa*				
Correct	461 (72.60)	218 (76.49)	243 (69.43)	0.048^*∗∗*^
Incorrect	174 (27.40)	67 (23.51)	107 (30.57)	
The microorganism that is the most common cause of nosocomial diarrhea in adults is *Clostridioides difficile*				
Correct	538 (84.72)	247 (86.67)	291 (83.14)	0.220
Incorrect	97 (15.28)	38 (13.33)	59 (16.86)	
Nosocomial staphylococcal infections are most often transmitted by the hands of medical personnel				
Correct	438 (68.98)	202 (70.88)	236 (67.43)	0.350
Incorrect	197 (31.02)	83 (29.12)	114 (32.57)	
If sepsis is suspected in a patient with a central venous line, blood should be collected from the central line and 2 separate peripheral lines for microbiological testing				
Correct	303 (47.72)	131 (45.96)	172 (49.14)	0.425
Incorrect	332 (52.28)	154 (54.04)	178 (50.88)	
The result of a microbiological urine test with an antibiogram is obtained after 2-3 days				
Correct	431 (67.87)	206 (72.28)	225 (64.29)	0.032^*∗∗*^
Incorrect	204 (32.13)	79 (27.72)	125 (35.71)	
The alarming agents (microorganisms) are methicillin-resistant *Staphylococcus aureus* (MRSA), *Escherichia coli* producing ESBL beta-lactamase, and vancomycin-resistant *Enterococcus faecium* (VRE)				
Correct	488 (76.85)	223 (78.25)	265 (75.71)	0.452
Incorrect	147 (23.15)	62 (21.75)	85 (24.29)	

^*∗*^Test *χ*^2^; ^*∗∗*^p < *α*; *α* = 0.05 statistical significance was found.

**Table 10:** Area III: ways to prevent nosocomial infections.

Components of area III/type of response	Together (*n* = 635; %)	Conservation ward (*n* = 285; %)	Treatment department (*n* = 350; %)	*p* value^*∗*^
Perioperative antibiotic prophylaxis is a short (usually 1 dose) administration of an antibiotic (usually cefazolin) just before surgery in a contaminated clean or clean field with a high risk of infection				
Correct	158 (24.88)	59 (20.70)	99 (28.29)	0.028^*∗∗*^
Incorrect	477 (75.12)	226 (79.30)	251 (71.71)	
Proper management of a patient who has a *Klebsiella pneumoniae* NDM strain detected in a rectal swab taken at hospital admission as part of screening is to isolate the patient until the end of the hospital stay in a separate room or cohort with patients with the same microorganism detected therefore, antibiotic therapy should be implemented				
Correct	214 (33.70)	106 (37.19)	108 (30.88)	0.093
Incorrect	421 (66.30)	179 (62.81)	242 (69.14)	
With the exception of contact with patients infected with *Clostridioides difficile*, the recommended method of hand decontamination during nursing procedures is hand disinfection using an alcohol-based hand rub				
Correct	463 (72.91)	207 (72.63)	256 (73.14)	0.885
Incorrect	172 (27.09)	78 (27.37)	94 (26.86)	
Nonsterile disposable gloves are used during contact with body fluids, excretions, and secretions of the patient and during all activities with an isolated patient who is colonized/infected with the alarm agent (microorganism)				
Correct	507 (79.84)	232 (81.40)	275 (78.57)	0.376
Incorrect	128 (20.16)	53 (18.60)	75 (21.43)	
When performing activities with a patient remaining in air-dust isolation, the nursing staff should be equipped with the following personal protective equipment: Gloves, protective apron, and filtering half-mask				
Correct	358 (56.38)	176 (61.75)	182 (52.00)	0.014^*∗∗*^
Incorrect	277 (43.62)	109 (38.25)	168 (48.00)	
Containers intended for medical waste with sharp ends or edges should be replaced when filled to a maximum of 2/3 of the volume, but at least once every 2-3 days				
Correct	522 (82.20)	235 (82.48)	287 (82.00)	0.881
Incorrect	113 (17.80)	50 (17.54)	63 (18.00)	
In order to properly disinfect the patient's skin before taking blood for laboratory tests, the alcohol agent should remain on the skin until it dries or according to the manufacturer's information on the preparation's packaging				
Correct	520 (81.89)	228 (80.00)	292 (83.43)	0.264
Incorrect	115 (18.11)	57 (20.00)	58 (16.57)	
The correct procedure after a needle injury, which was previously used for intravenous injection, is to wash the skin with plenty of lukewarm water and soap, do not squeeze the wound, do not stop bleeding, do not use alcohol-based disinfectants immediately after a cut or puncture, put on a sterile dressing, report a case of occupational exposure in the workplace				
Correct	584 (91.97)	267 (93.68)	317 (90.57)	0.151
Incorrect	51 (8.03)	18 (6.32)	33 (9.43)	

^*∗*^Test *χ*^2^; ^*∗∗*^*p* < *α*; *α* = 0.05 statistical significance was found.

## Data Availability

The data used in this study will be available upon request.
